# Cardiac Pacemaker Battery Discharge After External Electrical Cardioversion for Broad QRS Complex Tachycardia

**Published:** 2008-08-01

**Authors:** Martino Annamaria, Scapigliati Andrea, Casella Michela, Sanna Tommaso, Pelargonio Gemma, Dello Russo Antonio, Zamparelli Roberto, De Paulis Stefano, Bellocci Fulvio, Schiavello Rocco

**Affiliations:** 1 Institute of Cardiology, Department of Cardiovascular Medicine, Catholic University of the Sacred Heart, Rome, Italy; 2 Institute of Anesthesiology and Intensive Care, Department of Cardiovascular Medicine, Catholic University of the Sacred Heart, Rome, Italy

**Keywords:** Electric countershock, Defibrillation, Pacemaker, Electromagnetic interference, Ventricular arrhythmias

## Abstract

External electrical cardioversion or defibrillation may be necessary in patients with implanted cardiac pacemaker (PM) or implantable cardioverter defibrillator (ICD). Sudden discharge of high electrical energy employed in direct current (DC) transthoracic countershock may damage the PM/ICD system resulting in a series of possible device malfunctions. For this reason, when defibrillation or cardioversion must be attempted in a patient with a PM or ICD, some precautions should be taken, particularly in PM dependent patients, in order to prevent damage to the device. We report the case of a 76-year-old woman with a dual chamber PM implanted in the right subclavicular region, who received two consecutive transthoracic DC shocks to treat haemodynamically unstable broad QRS complex tachycardia after cardiac surgery performed with a standard sternotomic approach. Because of the sternal wound and thoracic drainage tubes together with the severe clinical compromise, the anterior paddle was positioned near the pulse generator. At the following PM test, a complete battery discharge was detected.

## Introduction

Pacemakers (PM) and implantable cardioverter defibrillators (ICDs) are currently being implanted in a great number of patients because of increasing indications; although these devices are very sophisticated, they may be affected by electromagnetic interference (EMI) generated by external sources like cellular phones, magnetic resonance imaging, radiotherapy, radiofrequency application during catheter ablation procedures, and transthoracic direct current (DC) shocks [1].

Despite PMs and ICDs have protective systems [2], the effect of EMI are unpredictable and include: software reprogramming; inappropriate inhibition; triggering or asynchronous pacing (V00 or VVI setting); acute loss of capture or chronic increase in stimulation thresholds of ventricular and atrial leads; and also damage of internal circuits [2-4]. When a DC shock must be performed in these patients, the defibrillator pads or paddles should not be placed close to or on top of the device. Most of PMs and ICDs are placed in the left subclavicular region; thus, the conventional defibrillator paddles position (left apical and right subclavicular position) is appropriate. However other sites can be used for device implantation (e.g. right subclavicular area) and they can be difficult to be detected in emergency situations. We report a case of sudden and complete PM battery discharge following two consecutive transthoracic DC shocks applied near the pulse generator in a patient with the device placed in the right pectoral region.

## Case report

A 76-year-old lady was admitted in the cardiac surgical department due to severe mitral valve regurgitation and moderate-severe tricuspid regurgitation. Because of brady-tachy syndrome, a dual chamber PM was previously implanted in 2002 and then replaced in  2006. The pulse generator was located in the right pectoral region and, via right subclavian vein, two unipolar leads were placed in right atrial appendage and in right ventricle apex respectively. Just before surgery, in the presence of normal sinus rhythm, the PM was programmed in V00 mode. The surgical procedure consisted of a mitral valve replacement with mechanical prosthesis and a tricuspid valvuloplasty with a standard sternotomic approach. As a routine measure, atrial and ventricular epicardial pacing electrodes for external pacing were positioned.

Afterwards a new PM test was performed with pacing setted in DDI at programmed lower rate of 85 beats/min, to avoid atrial arrhythmias, and with a long atrioventricular interval to promote spontaneous atrioventricular conduction.

During the night the patient, sedated and connected to mechanical ventilator, developed a broad QRS complex tachycardia episode (complex cycle 310 ms) associated with severe hypotension (arterial blood pressure: 70/20 mmHg). An emergency synchronized cardioversion using a 100 Joules DC shock was performed using a biphasic wave defibrillator, with an immediate return to sinus rhythm. After few seconds, a second DC shock (150 J) was necessary due to a relapse of the tachycardia. Lidocaine, 1 mg per Kg, was then administered to avoid tachycardia recurrences.

At the time of the episode, the patient chest was cluttered with a wide dressing, due to median sternotomy and thoracic drainage tubes. The severe clinical conditions advised staff against moving or rotating the patient on her side. Thus in both the DC shocks the right paddle was immediately placed laterally from the sternotomic surgical area, on the right pectoral region, above the right breast which occupied the axillary area, by the side of the implantation scar and near the pulse generator. The lateral paddle was positioned on the apex region.

Following the procedure, the patient showed a spontaneous sinus rhythm at 100 beats/min with correct inhibition of pacing. A new test of the implanted PM was performed showing a complete battery depletion of the pulse generator with end-of-life characteristics, while all the other programming parameters were unchanged (impedance, pacing and sensing thresholds of both leads were unchanged compared to pre DC shock test values). The device was replaced before patient discharge from the hospital.

## Discussion

Ventricular arrhythmias and broad QRS complex tachycardia can be a complication after cardiac surgery due to electrolyte disturbances, drugs and inotropes, cardioplegia, ischemia, perioperative myocardial infarction, or cardiogenic shock. Their treatment depends on the patient's haemodynamic status [5]. If signs of circulation are present but the patient is clinically unstable (e.g. impaired consciousness, chest pain, heart failure, hypotension, signs of shock), the tachycardia algorithm suggests to immediately attempt synchronised cardioversion (class I, level of evidence C). The DC shock must be synchronised with R wave of the ECG. It's better to start with 120-150 J biphasic energy levels for the initial shock and then increase if this fails (class IIa; level of evidence C) [5,6]. European Resuscitation Council 2005 Guidelines [7] describe  pads or paddles position for DC shock cardioversion and defibrillation in emergency situations. In case of ventricular arrhythmias the conventional sternal-apical position is recommended: the right (sternal) electrode should be placed to the right of the sternum, below the clavicle, and the left (apical) paddle must be placed in the left midaxillary line, approximately at the same level to V6 ECG electrode or to female breast. It is stated that this position should be clear of any breast tissue to minimize transthoracic impedance. Alternatively other acceptable pad positions are listed, in order to prevent damages when a medical implantable device is present: the biaxillary position, with each electrode, one on the right, and the other one on the left, on the lateral chest walls; the posterior-apical position with one electrode in the standard apical position and the other one on the right or left upper back; the anterior-posterior position with one electrode over the left precordium, and the other one on the back, just inferior to the left scapula. It does no matter which electrode (apex/sternum) is placed in either position [7]. All these placements have been shown to have equivalent and acceptable transthoracic impedance, with similar current flow [8] but there is no evidence from human studies that electrode position can affect outcome in case of shockable cardiac arrest [7].

A recent statement on emergency management of arrhythmias in patients with ICDs recommend to ensure a minimum distance of 5 cm between the edge of the paddle and the device. The conventional apical/right subclavicular paddles position is suggested, as most devices are implanted in the left subclavicular region and are usually readily apparent.

Other general precautions are suggested if the patient is haemodynamically stable (for example, to wait at least five minutes between two successive shocks so as to permit cooling of the diodes) and a specialist help is available to re-programme the device before shock delivery (Table 1). In any case, it is recommended to test PM function, pacing and sensing thresholds and leads impedence directly after cardioversion, at discharge, and about one month later [9,10].

In our case, the arrhythmia was associated with severe clinical conditions likely to progress to cardiac arrest and prompting a rapid response. The emergency situation and the time of arrhythmia presentation did not allow any specialist intervention and no immediate PM reprogramming before cardioversion was possible.

Alternative paddle positions, namely the posterior-apical and the anterior-posterior ones, were impossible to perform without moving or rotating the clinically unstable patient, adding further risks. Furthermore, the presence of sternal wound dressing and thoracic tubes allowed to position the right anterior paddle just on the right pectoral region, near the pulse generator located in the uncommon right site. Finally, the tachycardia relapse required a prompt second DC shock attempt without waiting the suggested interval between consecutive shocks. Patient safety, in case of post shock symptomatic bradycardia, was ensured by the external perioperative PM, even in case of implanted device damage.

We consider right paddle position as the likely cause of the battery failure as we found during PM interrogation the day after cardioversion. ECG failed to show any PM malfunction, indeed a correct PM inhibition was recorded. This malfunction required device replacement before patient discharge.

In order to prevent such damages, we suggest some further precautions that should be used in patients with a cardiac device implanted in the right pectoral region when other factors, as recent thoracic surgery, do not allow safe conventional paddle position.

Considering paddles placement, the biaxillary position would seem to be the most reliable as both the right and left paddle are far enough from any usual and unusual ICD/PM implantation site (left or right sublcavicular or abdominal regions). It does not need to move or rotate the patient. In high risk patients in which multiple arrhythmic episodes are possible, use of self-adhesive padds could be suggested. A problem with them, if they are left in situ, is the consequent shadow on chest X-ray which can add some difficulties in image interpretation; again, the biaxillary position could minimize this problem.

When a patient is recovering in a high dependency area where a DC shock delivery is likely, or if the patient has a PM dependent rhythm and is admitted in hospital, the exact perimeter of the pulse generator should be marked with a dermographic pencil or with a self adhesive plaster on the patient skin, because the external scar often doesn't reflect the exact position of the device. This mark can help a possible first responder to avoid hazardous paddles positioning.

Despite all precautions, risk of device malfunctioning after DC shock can not be nullified, requiring a strict specialist control of device functions after cardioversion or defibrillation.

## Conclusion

An increasing number of patients with an implanted PM or ICD could need a DC shock cardioversion or defibrillation for atrial or ventricular tachycardias. We present a case of life-threatening arrhythmia in which, after two consecutive DC shocks with a paddle positioned near the pulse generator, we noted a complete battery discharge of the device. Some suggestions to prevent such device failure are presented. In emergency situation first responder must be aware of the exact device site. Generic biaxillary paddle position can not be enough to reduce the risk of device damage. We suggest that patients with implanted ICD/PM, especially those at risk of developing malignant arrhythmias, must be checked at admission in hospital to highlight the actual device position. This should be marked clearly to allow safe DC shock delivery, in case of necessity.

## Figures and Tables

**Table 1:**
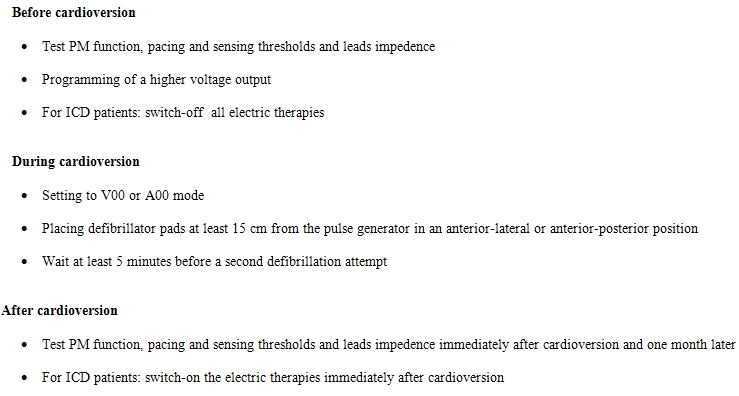
PM/ICD checklist in case of cardioversion
